# Supplementary material for: Modeling glioblastoma heterogeneity as a dynamic network of cell states
Source: Mol Syst Biol. 2021 Sep 16;17(9):e10105. doi: 10.15252/msb.202010105 (PMC8444284; doi:10.15252/msb.202010105)
Supplement: Supplementary file 5 — Source Data for Figure 3 [file MSB-17-e10105-s001.zip › Figure3A_sourcedata/GSEA_3065/hallmarks_state3.GseaPreranked.1623416274744/HALLMARK_MTORC1_SIGNALING.html]

Details for gene set HALLMARK\_MTORC1\_SIGNALING[GSEA]

|  || Dataset | state3 |
| Phenotype | NoPhenotypeAvailable |
| Upregulated in class | na\_pos |
| GeneSet | HALLMARK\_MTORC1\_SIGNALING |
| Enrichment Score (ES) | 0.38589945 |
| Normalized Enrichment Score (NES) | 1.6465966 |
| Nominal p-value | 0.0 |
| FDR q-value | 0.019719224 |
| FWER p-Value | 0.049 |
Table: GSEA Results Summary

  

Fig 1: Enrichment plot: HALLMARK\_MTORC1\_SIGNALING      
 Profile of the Running ES Score & Positions of GeneSet Members on the Rank Ordered List

  

| PROBE | GENE SYMBOL | GENE\_TITLE | RANK IN GENE LIST | RANK METRIC SCORE | RUNNING ES | CORE ENRICHMENT || 1 | HSP90B1 |  |  | 7 | 0.563 | 0.0256 | Yes |
| 2 | CANX |  |  | 27 | 0.479 | 0.0458 | Yes |
| 3 | CALR |  |  | 30 | 0.473 | 0.0678 | Yes |
| 4 | CD9 |  |  | 32 | 0.473 | 0.0899 | Yes |
| 5 | SQLE |  |  | 39 | 0.457 | 0.1106 | Yes |
| 6 | HSPA5 |  |  | 43 | 0.447 | 0.1312 | Yes |
| 7 | HMGCR |  |  | 77 | 0.369 | 0.1447 | Yes |
| 8 | SCD |  |  | 108 | 0.340 | 0.1571 | Yes |
| 9 | FKBP2 |  |  | 127 | 0.319 | 0.1700 | Yes |
| 10 | ACSL3 |  |  | 130 | 0.319 | 0.1847 | Yes |
| 11 | FADS2 |  |  | 153 | 0.309 | 0.1967 | Yes |
| 12 | RPN1 |  |  | 178 | 0.296 | 0.2078 | Yes |
| 13 | SEC11A |  |  | 205 | 0.281 | 0.2179 | Yes |
| 14 | RDH11 |  |  | 212 | 0.277 | 0.2302 | Yes |
| 15 | CTSC |  |  | 226 | 0.272 | 0.2414 | Yes |
| 16 | DHCR24 |  |  | 244 | 0.265 | 0.2519 | Yes |
| 17 | HMGCS1 |  |  | 267 | 0.254 | 0.2612 | Yes |
| 18 | FADS1 |  |  | 331 | 0.234 | 0.2649 | Yes |
| 19 | ELOVL5 |  |  | 450 | 0.209 | 0.2610 | Yes |
| 20 | MCM4 |  |  | 482 | 0.201 | 0.2668 | Yes |
| 21 | LDLR |  |  | 498 | 0.199 | 0.2744 | Yes |
| 22 | M6PR |  |  | 520 | 0.196 | 0.2811 | Yes |
| 23 | SLC2A1 |  |  | 587 | 0.188 | 0.2823 | Yes |
| 24 | SERPINH1 |  |  | 588 | 0.188 | 0.2911 | Yes |
| 25 | ACLY |  |  | 624 | 0.183 | 0.2956 | Yes |
| 26 | P4HA1 |  |  | 649 | 0.179 | 0.3012 | Yes |
| 27 | DHCR7 |  |  | 669 | 0.176 | 0.3072 | Yes |
| 28 | PLOD2 |  |  | 683 | 0.173 | 0.3138 | Yes |
| 29 | BUB1 |  |  | 717 | 0.168 | 0.3179 | Yes |
| 30 | TMEM97 |  |  | 740 | 0.165 | 0.3231 | Yes |
| 31 | ATP2A2 |  |  | 792 | 0.158 | 0.3246 | Yes |
| 32 | SDF2L1 |  |  | 801 | 0.157 | 0.3310 | Yes |
| 33 | USO1 |  |  | 810 | 0.157 | 0.3374 | Yes |
| 34 | GLA |  |  | 816 | 0.156 | 0.3442 | Yes |
| 35 | HSPA4 |  |  | 824 | 0.155 | 0.3506 | Yes |
| 36 | PHGDH |  |  | 828 | 0.155 | 0.3575 | Yes |
| 37 | SSR1 |  |  | 851 | 0.152 | 0.3621 | Yes |
| 38 | EBP |  |  | 897 | 0.149 | 0.3638 | Yes |
| 39 | SC5D |  |  | 914 | 0.147 | 0.3689 | Yes |
| 40 | GMPS |  |  | 954 | 0.142 | 0.3710 | Yes |
| 41 | CYP51A1 |  |  | 976 | 0.140 | 0.3751 | Yes |
| 42 | SLC2A3 |  |  | 1026 | 0.136 | 0.3758 | Yes |
| 43 | XBP1 |  |  | 1036 | 0.135 | 0.3811 | Yes |
| 44 | ELOVL6 |  |  | 1050 | 0.134 | 0.3859 | Yes |
| 45 | PSMD14 |  |  | 1227 | 0.120 | 0.3710 | No |
| 46 | SLC37A4 |  |  | 1251 | 0.118 | 0.3739 | No |
| 47 | TFRC |  |  | 1304 | 0.115 | 0.3732 | No |
| 48 | SLC7A5 |  |  | 1394 | 0.109 | 0.3679 | No |
| 49 | NUP205 |  |  | 1397 | 0.108 | 0.3728 | No |
| 50 | ACACA |  |  | 1412 | 0.108 | 0.3762 | No |
| 51 | LGMN |  |  | 1637 | 0.095 | 0.3546 | No |
| 52 | MCM2 |  |  | 1762 | 0.088 | 0.3442 | No |
| 53 | SLC1A4 |  |  | 2003 | 0.076 | 0.3199 | No |
| 54 | BCAT1 |  |  | 2034 | 0.075 | 0.3199 | No |
| 55 | TM7SF2 |  |  | 2168 | 0.070 | 0.3076 | No |
| 56 | IDI1 |  |  | 2285 | 0.065 | 0.2971 | No |
| 57 | IDH1 |  |  | 2376 | 0.062 | 0.2896 | No |
| 58 | HSPA9 |  |  | 2404 | 0.060 | 0.2893 | No |
| 59 | IFRD1 |  |  | 2444 | 0.059 | 0.2875 | No |
| 60 | CXCR4 |  |  | 2543 | 0.055 | 0.2787 | No |
| 61 | NAMPT |  |  | 2583 | 0.054 | 0.2767 | No |
| 62 | CCNF |  |  | 2588 | 0.054 | 0.2787 | No |
| 63 | PSMA4 |  |  | 2605 | 0.053 | 0.2794 | No |
| 64 | PSMD12 |  |  | 2658 | 0.051 | 0.2757 | No |
| 65 | SLC7A11 |  |  | 2682 | 0.051 | 0.2754 | No |
| 66 | HSPD1 |  |  | 2700 | 0.050 | 0.2758 | No |
| 67 | EDEM1 |  |  | 2724 | 0.049 | 0.2754 | No |
| 68 | VLDLR |  |  | 2734 | 0.049 | 0.2767 | No |
| 69 | SKAP2 |  |  | 2771 | 0.048 | 0.2747 | No |
| 70 | RRM2 |  |  | 2835 | 0.046 | 0.2695 | No |
| 71 | ADD3 |  |  | 2929 | 0.043 | 0.2607 | No |
| 72 | ERO1A |  |  | 3035 | 0.040 | 0.2504 | No |
| 73 | ADIPOR2 |  |  | 3051 | 0.040 | 0.2505 | No |
| 74 | IMMT |  |  | 3080 | 0.039 | 0.2491 | No |
| 75 | PITPNB |  |  | 3092 | 0.039 | 0.2496 | No |
| 76 | INSIG1 |  |  | 3136 | 0.038 | 0.2463 | No |
| 77 | PSMC4 |  |  | 3328 | 0.032 | 0.2256 | No |
| 78 | TOMM40 |  |  | 3348 | 0.032 | 0.2249 | No |
| 79 | AURKA |  |  | 3503 | 0.028 | 0.2083 | No |
| 80 | IGFBP5 |  |  | 3555 | 0.027 | 0.2036 | No |
| 81 | GSK3B |  |  | 3606 | 0.025 | 0.1989 | No |
| 82 | POLR3G |  |  | 3660 | 0.024 | 0.1939 | No |
| 83 | SLC6A6 |  |  | 3732 | 0.022 | 0.1866 | No |
| 84 | SHMT2 |  |  | 3781 | 0.021 | 0.1820 | No |
| 85 | PIK3R3 |  |  | 3885 | 0.018 | 0.1709 | No |
| 86 | GOT1 |  |  | 3908 | 0.017 | 0.1691 | No |
| 87 | CDC25A |  |  | 4065 | 0.013 | 0.1516 | No |
| 88 | BTG2 |  |  | 4070 | 0.013 | 0.1517 | No |
| 89 | PGM1 |  |  | 4098 | 0.013 | 0.1492 | No |
| 90 | ASNS |  |  | 4228 | 0.009 | 0.1345 | No |
| 91 | PPA1 |  |  | 4255 | 0.008 | 0.1319 | No |
| 92 | MAP2K3 |  |  | 4277 | 0.007 | 0.1298 | No |
| 93 | STIP1 |  |  | 4577 | -0.001 | 0.0950 | No |
| 94 | GBE1 |  |  | 4604 | -0.001 | 0.0920 | No |
| 95 | GGA2 |  |  | 4639 | -0.002 | 0.0882 | No |
| 96 | DDX39A |  |  | 4706 | -0.004 | 0.0807 | No |
| 97 | EEF1E1 |  |  | 4876 | -0.008 | 0.0613 | No |
| 98 | UNG |  |  | 4979 | -0.010 | 0.0499 | No |
| 99 | PPP1R15A |  |  | 4983 | -0.010 | 0.0501 | No |
| 100 | NMT1 |  |  | 4988 | -0.011 | 0.0501 | No |
| 101 | TBK1 |  |  | 4992 | -0.011 | 0.0503 | No |
| 102 | DDIT4 |  |  | 5025 | -0.012 | 0.0471 | No |
| 103 | MTHFD2L |  |  | 5124 | -0.014 | 0.0363 | No |
| 104 | PSMC6 |  |  | 5139 | -0.014 | 0.0354 | No |
| 105 | PLK1 |  |  | 5235 | -0.017 | 0.0251 | No |
| 106 | BHLHE40 |  |  | 5331 | -0.019 | 0.0149 | No |
| 107 | NFKBIB |  |  | 5335 | -0.019 | 0.0155 | No |
| 108 | NFYC |  |  | 5374 | -0.020 | 0.0120 | No |
| 109 | RIT1 |  |  | 5453 | -0.023 | 0.0040 | No |
| 110 | LTA4H |  |  | 5492 | -0.024 | 0.0007 | No |
| 111 | PSMA3 |  |  | 5498 | -0.024 | 0.0012 | No |
| 112 | RRP9 |  |  | 5569 | -0.026 | -0.0057 | No |
| 113 | GPI |  |  | 5670 | -0.029 | -0.0160 | No |
| 114 | UCHL5 |  |  | 5838 | -0.034 | -0.0339 | No |
| 115 | GSR |  |  | 5892 | -0.035 | -0.0384 | No |
| 116 | STC1 |  |  | 5905 | -0.036 | -0.0381 | No |
| 117 | PSMG1 |  |  | 5991 | -0.039 | -0.0462 | No |
| 118 | TXNRD1 |  |  | 6009 | -0.040 | -0.0463 | No |
| 119 | RPA1 |  |  | 6115 | -0.044 | -0.0565 | No |
| 120 | ACTR3 |  |  | 6122 | -0.044 | -0.0551 | No |
| 121 | ACTR2 |  |  | 6282 | -0.049 | -0.0713 | No |
| 122 | FDXR |  |  | 6438 | -0.054 | -0.0868 | No |
| 123 | ALDOA |  |  | 6448 | -0.054 | -0.0853 | No |
| 124 | GTF2H1 |  |  | 6499 | -0.056 | -0.0885 | No |
| 125 | NUFIP1 |  |  | 6555 | -0.059 | -0.0922 | No |
| 126 | PSMC2 |  |  | 6627 | -0.062 | -0.0975 | No |
| 127 | MTHFD2 |  |  | 6745 | -0.068 | -0.1079 | No |
| 128 | CYB5B |  |  | 6751 | -0.068 | -0.1053 | No |
| 129 | PFKL |  |  | 6771 | -0.070 | -0.1043 | No |
| 130 | PSMB5 |  |  | 6794 | -0.071 | -0.1035 | No |
| 131 | ETF1 |  |  | 6805 | -0.071 | -0.1013 | No |
| 132 | COPS5 |  |  | 6815 | -0.072 | -0.0990 | No |
| 133 | SRD5A1 |  |  | 6841 | -0.073 | -0.0985 | No |
| 134 | DDIT3 |  |  | 6918 | -0.077 | -0.1038 | No |
| 135 | UFM1 |  |  | 6947 | -0.078 | -0.1034 | No |
| 136 | PNO1 |  |  | 6983 | -0.079 | -0.1037 | No |
| 137 | ATP6V1D |  |  | 6994 | -0.080 | -0.1011 | No |
| 138 | PSMD13 |  |  | 7101 | -0.087 | -0.1094 | No |
| 139 | PGK1 |  |  | 7115 | -0.088 | -0.1068 | No |
| 140 | HMBS |  |  | 7199 | -0.093 | -0.1121 | No |
| 141 | PSME3 |  |  | 7207 | -0.094 | -0.1085 | No |
| 142 | ENO1 |  |  | 7257 | -0.097 | -0.1096 | No |
| 143 | GLRX |  |  | 7283 | -0.099 | -0.1079 | No |
| 144 | QDPR |  |  | 7488 | -0.116 | -0.1262 | No |
| 145 | CCNG1 |  |  | 7558 | -0.123 | -0.1285 | No |
| 146 | PSPH |  |  | 7577 | -0.125 | -0.1247 | No |
| 147 | DHFR |  |  | 7593 | -0.127 | -0.1205 | No |
| 148 | PSAT1 |  |  | 7634 | -0.131 | -0.1190 | No |
| 149 | HPRT1 |  |  | 7679 | -0.137 | -0.1178 | No |
| 150 | CACYBP |  |  | 7708 | -0.141 | -0.1144 | No |
| 151 | ATP5MC1 |  |  | 7743 | -0.145 | -0.1116 | No |
| 152 | EIF2S2 |  |  | 7758 | -0.147 | -0.1064 | No |
| 153 | SYTL2 |  |  | 7797 | -0.151 | -0.1037 | No |
| 154 | G6PD |  |  | 7860 | -0.160 | -0.1034 | No |
| 155 | HSPE1 |  |  | 7918 | -0.170 | -0.1021 | No |
| 156 | RAB1A |  |  | 7920 | -0.170 | -0.0942 | No |
| 157 | TUBG1 |  |  | 7966 | -0.179 | -0.0910 | No |
| 158 | YKT6 |  |  | 8147 | -0.214 | -0.1019 | No |
| 159 | UBE2D3 |  |  | 8174 | -0.221 | -0.0946 | No |
| 160 | SQSTM1 |  |  | 8176 | -0.221 | -0.0844 | No |
| 161 | SERP1 |  |  | 8229 | -0.235 | -0.0794 | No |
| 162 | TCEA1 |  |  | 8230 | -0.235 | -0.0684 | No |
| 163 | ARPC5L |  |  | 8342 | -0.270 | -0.0687 | No |
| 164 | PDAP1 |  |  | 8369 | -0.280 | -0.0585 | No |
| 165 | MLLT11 |  |  | 8489 | -0.335 | -0.0567 | No |
| 166 | GAPDH |  |  | 8615 | -0.418 | -0.0516 | No |
| 167 | PPIA |  |  | 8626 | -0.432 | -0.0325 | No |
| 168 | CDKN1A |  |  | 8657 | -0.461 | -0.0144 | No |
| 169 | TPI1 |  |  | 8709 | -0.538 | 0.0049 | No |
Table: GSEA details [plain text format]

  

Fig 2: HALLMARK\_MTORC1\_SIGNALING: Random ES distribution      
 Gene set null distribution of ES for **HALLMARK\_MTORC1\_SIGNALING**
